# Identification of prognostic genes and gene sets for early-stage non-small cell lung cancer using bi-level selection methods

**DOI:** 10.1038/srep46164

**Published:** 2017-04-07

**Authors:** Suyan Tian, Chi Wang, Howard H. Chang, Jianguo Sun

**Affiliations:** 1 Center for Applied Statistical Research, School of Mathematics, Jilin University, 2699 Qianjin Street, Changchun, Jilin, 130012, China; 2 Division of Clinical Research, First Hospital of Jilin University, 71 Xinmin Street, Changchun, Jilin, 130021, China; 3 Department of Biostatistics, Markey Cancer Center, University of Kentucky, 800 Rose St., Lexington, KY, 40536, USA; 4 Department of Biostatistics and Bioinformatics, Rollins School of Public Health, Emory University, 1518 Clifton Road NE, Atlanta, GA, 30322, USA; 5 Department of Statistics, University of Missouri, 146 Middlebush Hall, Columbia, MO, 65211, USA

## Abstract

In contrast to feature selection and gene set analysis, bi-level selection is a process of selecting not only important gene sets but also important genes within those gene sets. Depending on the order of selections, a bi-level selection method can be classified into three categories – forward selection, which first selects relevant gene sets followed by the selection of relevant individual genes; backward selection which takes the reversed order; and simultaneous selection, which performs the two tasks simultaneously usually with the aids of a penalized regression model. To test the existence of subtype-specific prognostic genes for non-small cell lung cancer (NSCLC), we had previously proposed the Cox-filter method that examines the association between patients’ survival time after diagnosis with one specific gene, the disease subtypes, and their interaction terms. In this study, we further extend it to carry out forward and backward bi-level selection. Using simulations and a NSCLC application, we demonstrate that the forward selection outperforms the backward selection and other relevant algorithms in our setting. Both proposed methods are readily understandable and interpretable. Therefore, they represent useful tools for the researchers who are interested in exploring the prognostic value of gene expression data for specific subtypes or stages of a disease.

Feature selection, where the primary objective is selection of individual relevant variables, such as genes associated with a phenotype of interest, is an important topic in the field of bioinformatics. Because gene expression profiles exhibit grouped structure with genes inside each group being highly correlated to each other, gene set analysis has become popular and widespread over the last decade^1^,^2^. The goal of a gene set analysis method is to examine the association of a gene set with the phenotype of interest, meaning the selection of relevant gene sets. Both gene and gene set selections lead to more parsimonious final models with better predictive performance and more meaningful biological interpretation.

In contrast to feature selection and gene set analysis, bi-level selection is a process of selecting relevant features at two levels, as its name implies^3^. Bi-level selection is motivated by the fact that some genes within a gene set may be unrelated to the phenotype of interest, although the gene set as a whole is involved in the biological process. Since a bi-level selection analysis is interested in selecting not only important groups/gene sets but also those important members/genes inside those groups/gene sets, a feature may be thus referred to as either a gene set or a gene here.

Based on the order of selection on genes and gene sets, bi-level selection methods can be classified into three categories: (1) the forward selection method which first selects relevant gene sets followed by the selection of relevant individual genes, e.g.,^4^; (2) the backward selection method such as^5^,^6^,^7^ which takes the reversed order; and (3) the simultaneous selection method which selects important gene sets and genes at the same time, usually by the means of adding a penalty term which penalizes all genes inside the same group similarly, e.g.,^3^,^8^. The simultaneous selection method using a penalized regression model is more statistically sophisticated, compared to the first two methods. Here we focus on the forward and backward selection methods due to their ease in implementation and thus wide utilization.

Even though both the forward and backward selection methods can implement two level selections, their relative emphases on these two levels may differ. Nevertheless, their common drawback is that the small number of selected features in the first step may lead to challenges in performing the second step. Intuitively, the forward method is superior to the backward method because the extraction of core genes before the identification of important gene sets may screen out the gene sets whose members have subtle individual effects but their coordinated effects being significant when taken together.

Non-small cell lung cancer (NSCLC) is a leading cause of cancer deaths in many countries^9^. Adenocarcinoma (AC) and squamous cell carcinoma (SCC) are two major histological subtypes of NSCLC. There is increasing evidence to support that AC and SCC differ in the composition of genes and molecular characteristics^10^. Therefore, they should be regarded as two distinct diseases and treated with different treatment strategies.

Currently, treatment choices for these two subtypes are very homogeneous, mainly depending on the stage at which the cancer is diagnosed. It is becoming critical to evaluate the risk profiles of patients using a reliable gene/gene set signature. Given the fundamental differences between AC and SCC, the genes/gene sets associated with recurrence and survival rates for each histology subtype are expected to be different^11^,^12^,^13^. To test the existence of subtype-specific prognostic genes, two statistical methods were proposed previously by us^11^,^12^. For example, the so-called Cox-filter model^11^ fits a Cox model using gene, subtype, and their interaction term as covariates for each gene. After filtering and excluding insignificant genes, the Cox-filter model identifies subtype-specific prognostic genes (discussed in detail in the Methods section). However, the Cox-filter method does not take the grouping structures among genes into account, and thus selects genes based on the strength of their individual effects and introduces false positives.

In this study, we extend the Cox-filter model to two bi-level selection methods — one forward method and one backward method — by constructing patients’ risk profiles at the gene set level using the sign average method^6^,^14^. The two Cox-filter extensions are then applied to NSCLC data to investigate if they can alleviate the limitations of the original Cox-filter model.

## Results

### Simulated Data

To examine the characteristics of our proposed procedures, and to explore if both extensions can alleviate the disadvantages of the Cox-filter method, we used actual expression values of the microarray data to conduct simulations. We used the first 100 gene sets in the c5 category of the Molecular Signatures Database and then randomly selected 4 genes – ARRB1, COPA, ECE2, and SMAD4 – to be prognostic markers. Among them, COPA, ECE2 and SMAD4 belong to the same gene set while ARRB1 is inside a different gene set.

Similar to the simulation setting in our previous study, we considered two extreme cases: (1) the mutually exclusive prognostic genes case in which AC and SCC have completely distinct sets of survival-relevant genes, and (2) no subtype-specific prognostic genes case in which AC and SCC share identical prognostic markers. In other words, for the first case, β_AC_s for genes 1 and 3 while β_scc_s for genes 2 and 4 are set to non-zero values. The hazard functions are specified as:


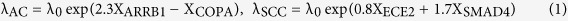


the survival time for each patient was simulated via a Cox-exponential distribution^15^ according to the above hazard functions, and the censoring rate was set at 30%.

For the other case, the hazard function is identical for both AC and SCC subtypes:


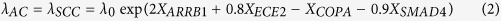


this means that all β_AC_s and β_scc_s for genes 1–4 are expected to have non-zero coefficients. For both scenarios, we simulated 50 datasets and applied our proposed procedures to these replicates. The percentages of the causal genes being selected over the 50 replicates are summarized in Table 1 for simulation 1 and in Table 2 for simulation 2, respectively. In both tables, the C-statistics and the Rand Index at the gene level and their standard errors are also provided, on which the comparison between the bi-level selection methods and other relevant algorithms, namely, the Cox-filter model^11^, the Cox-TGDR method^12^, and a separate LASSO^16^ for each subtype, was made.

In both simulations, the forward Cox-filter was superior to the backward Cox-filter in terms of the frequencies of identifying the true causal genes and the sizes of the final models. Compared with the original Cox-filter method, which selects genes simply based on individual effects, both the forward Cox-filter method and the backward Cox-filter method are more likely to select those genes with small individual effects but significant coordinated effects together with other genes in the same gene set, i.e., COPA, ECE2, and SMAD2. Nevertheless, as a filter method to select genes, the model parsimony (i.e., the number of genes in the resulting prognostic gene signature) of these two proposed methods remains unsatisfactory.

As a reference, the percentages of these four causal genes being selected under a random guess model by both forward Cox-filter and backward Cox-filter are presented in Table 3. Under the null model, almost all percentages of these genes being selected are zeros, suggesting the high frequencies obtained by both bi-level selection methods are impossible to occur by chance alone.

Overall, the forward Cox-filter method has the best performance when the C-statistics and the Rand Index are considered together. The backward Cox-filter method shows no such an overall superiority. For instance, in the first simulation the backward Cox-filter method has the lowest Rand index (15.87%) among these 5 algorithms but a very large standard error (5.04%) for the SCC subtype.

### Real World Data

In this application, we first trained on the NSCLC microarray data using both proposed methods and validated the predictive performance of the resulting gene signatures using the RNA-seq data as a test set. Then we reversed the order of the training set and the test set. Lastly, we applied three relevant methods—the Cox-filter method and the Cox-TGDR method proposed by us^11^,^12^ to test the existence of subtype-specific prognostic genes, and LASSO for survival analysis^17^ – to the NSCLC microarray data and compared both proposed bi-level selection methods and the three relevant methods. The performance statistics, i.e., the C-statistics calculated by applying the prognostic signatures on the test set and the Rand Index at the gene and gene set levels using 10-fold cross-validations, and their standard errors obtained using bootstrapped samples are presented in Table 4.

The forward Cox-filter method outperformed the three other relevant feature selection methods and the backward Cox-filter method. Of note, a non-statistically different C-index value of 52.8% (standard error: 2.75%) from a random guess model was obtained by the backward selection method for the SCC subtype. It is consistent with our expectation that the performance of the backward selection method is inferior to that of the forward selection method and the simulation results. However, we don’t exclude the likelihood that the backward selection method may be optimal for some specific data types or structures.

To verify if the resulting prognostic signatures are confounded by other known variables, i.e., age, sex, and smoking status, a multiple Cox regression model was fitted using the risk scores estimated from the resulting prognostic signature and these clinical variables as covariates. The results are presented in Table 5. Based on the most significant p-values for the prognostic signatures, it is concluded that the resulting prognostic signatures possess adequate prognostic capacity in predicting the survival of NSCLC patients.

**Figure 1:**
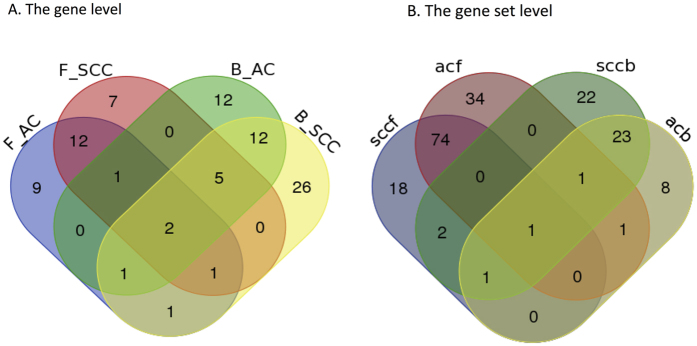
Venn diagrams showing the overlaps between the selected gene/gene sets for AC and SCC. (**A**) **At the gene level:** F_AC: the selected genes by the forward method for AC; F_SCC: the selected genes by the forward method for SCC; B_AC: the selected genes by the backward method for AC; B_SCC: the selected genes by the backward method for SCC; (**B**) **At the gene set level:** sccf: the selected gene sets by the forward method for SCC; acf: the selected gene sets by the forward method for AC; sccb: the selected gene sets by the backward method for SCC; acb: the selected gene sets by the backward method for AC.

Moreover, as shown by the Venn-diagrams in Fig. 1, the selected gene sets and genes using the forward method and the backward method share no or limited overlap. This finding indicates that the focuses of the two methods might be distinct. While for the NSCLC application both methods tend to improve the pathway-level and gene-level stabilities, it appears that the increment in pathway-level stability for the forward Cox-filter method is dramatically larger than the gene-level stability. In contrast, the backward Cox-filter method does not possess this feature. Such a pattern has been overlooked by previous work in which researchers only illustrate when a method accounts for pathway knowledge, its stabilities at both gene and gene set levels may be improved.

In contrast, the overlaps between respective gene sets and genes for AC and SCC using either the forward method or the backward method are substantially larger, implying that there might exist more pan genes or gene sets for both subtypes than subtype-specific ones. Although we emphasize the importance of those subtype specific gene/gene sets, the critical role played by those pan gene/gene sets cannot be denied as well.

## Conclusions

Using simulated data and a real-world application, we demonstrate that the forward Cox-filter method outperforms relevant algorithms under consideration as well as its backward counterpart in terms of gene-level and pathway-level stabilities and performance statistics. Given that there are numerous pathway analysis methods and feature selection algorithms, the forward Cox-filter method cannot be the “supermodel”—the optimal model for every expression data. Furthermore, as a bi-level selection method, the performance of the forward Cox-filter method relies on the quality of pathway knowledge. More specifically, these two proposed bi-level selection methods make an implicit assumption that all genes within a specific gene set shall co-function together to influence the phenotype of interest, which may not be true for all diseases.

We emphasize that we do not make any recommendation on the clinical utilization of the resulting prognostic gene signatures or gene set signatures. The primary objective of this study is to introduce the two bi-level selection methods. These two methods are easily implementable and readily interpretable, even for a biologist or clinician since it builds upon the Cox-models^18^ and the signed averages of expression values over a gene set or selected subset^14^. Therefore, they are easily accessible for researchers who are interested in exploring the prognostic value of gene expression data for specific subtypes or stages of a disease. As the first effort to address the issue of identifying both subtype-specific prognostic gene sets and genes for the early-stage NSCLC patients while accounting for the pathway information, the proposed methods may spark interest in this research area and propel the development of more advanced statistical methods.

## Methods

### Experimental Data

The RNA-Seq data for those patients at early histology stages (stages I and II) were downloaded from The Cancer Genome Atlas (https://tcga-data.nci.nih.gov/tcga/). By restricting the patients to those at early stages and being adjuvant treatment naïve with survival information, 70 AC and 55 SCC subjects remained.

The microarray data used were the experiments of GSE50081 in the GEO repository. The chips in this dataset were hybridized on the Affymetrix HGU133Plus 2.0 platform. After deleting those samples with ambiguous labels, 127 AC and 42 SCC patients were included in this study.

#### Gene Sets

Gene sets were downloaded from the **Molecular Signatures Database** (MSigDB)^19^. In this study, we considered only the c5 category. The current version (version 5.1) of the MSigDB c5 category includes 1554 gene sets annotated by the Gene Ontology (GO)^20^ terms.

### Pre-processing Procedures

Raw data (CEL files) of the microarray data set were downloaded from the GEO repository. Expression values were obtained using the fRMA algorithm^21^, and were normalized using quantile normalization. Then moderated t-tests using limma^22^ were carried out to identify the differentially expressed genes (DEGs) between SCC and AC in the microarray data set, and those non-DEGs with the false discovery rate (FDR) > 0.05 were filtered out. To deal with multiple probe sets matched to one specific gene, the one with the largest fold change was retained.

For the RNA-seq data, Counts-per-million (CPM) values were calculated and log_2_ transformed by the Voom function^23^ in R limma package^22^. The downstream analysis was conducted upon the 2569 genes inside microarray data, RNA-seq data, and the annotated gene sets.

### Statistical Methods

#### Cox-filter for subtype-specific prognosis

The Cox-filter method proposed by us^11^ is used to identify genes informative of survival rate for AC/SCC histology subtypes. In this method, a Cox model is fitted on each gene, and the hazard function of patient *i* for gene *g (g = *1,…,p) at time point t is given by,


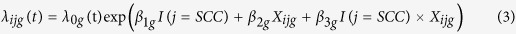


here, X_ij_ = (X_ij1_,…,X_ijp_)^T^ represent expression values for the p genes under consideration and λ_0g_(t) is an unknown baseline hazard function at time point t. I(j=SCC) is an indicator, taking the value of 1 if the histology subtype *j* of patient *i* is SCC. Otherwise, its value is 0 if the histology subtype of this patient is AC. Both β_2g_ and β_3g_ are the parameters of interest, with β_2g_ representing the change in log hazard rate associated with 1-unit increase in the actual expression value of gene *g* among AC, and β_3g_ representing the additional change in log hazard rate associated with the SCC subtype. The values of β_ACg_, i.e., β_2g_, and β_SCCg_, i.e., β_2g_ + β_3g_, determine if subtype-specific prognostic genes do exist. For example, β_ACg_≠0 but β_SCCg_ = 0 corresponds to an AC-specific gene and β_SCCg_≠0 but β_ACg  _=0 corresponds to an SCC-specific gene. In practice, we may also be interested in the scenario of both β_ACg_ and β_SCCg_ having different non-zero values.

#### Sign average

After fitting the Cox-filter model for each gene and obtaining estimated β_ACg_ and β_SCCg_ for each gene, we take the membership of genes into consideration and then summarize a patient’s risk profile as the sign average of his/her expression values over all genes/selected genes inside each specific gene set.

Specifically for each subtype, all genes/selected genes inside this gene set are classified into two groups according to the signs of their estimated effects in the above equation — the hazardous group H and the preventive group P. In the first group, genes with increased expression that are associated with higher hazard are included. In contrast, genes for which an increment in expression reduces hazard are classified into the second group. Since one gene may be hazardous in one subtype while preventive in the other, we introduce two sets of notations for the AC and SCC patients, i.e., H^AC^ and P^AC^ for AC patients, and H^SCC^ and P^SCC^ for SCC patients.

Then the sign average Z_ijk_ for patient i of subtype j (either AC or SCC) in gene set k is calculated as


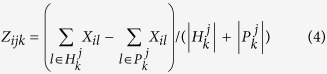


here 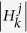 is the number of genes inside gene set 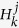. and X_il_ represents the gene expression value of gene *l* (l includes all genes belong to one specific gene set) for patient *i*. By taking the difference between the sum of expression values of all genes inside the hazardous group H and that of all genes inside the preventive group P and then dividing by the total number of genes/selected genes, Z_ijk_ is essentially the signed average of expression values over genes under consideration inside gene set k.

#### Extensions to the Cox-filter model for bi-selection

In order to implement bi-level selection with the Cox model in Eq. 3, we apply it twice in a either forward or backward way. Correspondingly, we refer them as the forward Cox-filter method and the backward Cox-filter method.

#### Forward Cox-filter

In the forward Cox-filter extension, the risk profiles for patient *i* are first calculated over all genes inside the specific gene set k, denoted as Z_ijk_. Here, Z_ij_ = (Z_ij1_,…,Z_ijK_)^T^ is a K-length vector representing the gene set level risk profiles for patient *i* over K gene sets under consideration.

Replacing genes by gene sets, then the Cox-filter model is fitted again. For one specific gene set k (k = 1,2,…,K, here K is the number of gene sets under consideration), the Cox-filter model may be expressed as,


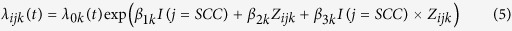


where Z_ijk_ is the sign average obtained in Eq.4, representing the expression level of gene set k for patient i. After filtering out those insignificant gene sets for whose adjusted p-values (using the procedure) are larger than a pre-determined cut-off, the significant levels of genes inside the identified gene sets are determined on the basis of the adjusted p-values of the Cox-filter models in Eq. 3.

#### Backward Cox-filter

Taking the reversed selection orders, the backward Cox-filter extension first selects candidate genes based on the adjusted p-values of the Cox-filter models in Eq. 3 and then calculates the risk profiles over those selected genes for each gene set. Lastly, the significant levels of gene sets are determined using the adjusted p-values of the Cox models in Eq. 5. Correspondingly, the final selected genes are those involved in the first step and also contained inside those significant gene sets.

**Figure 2:**
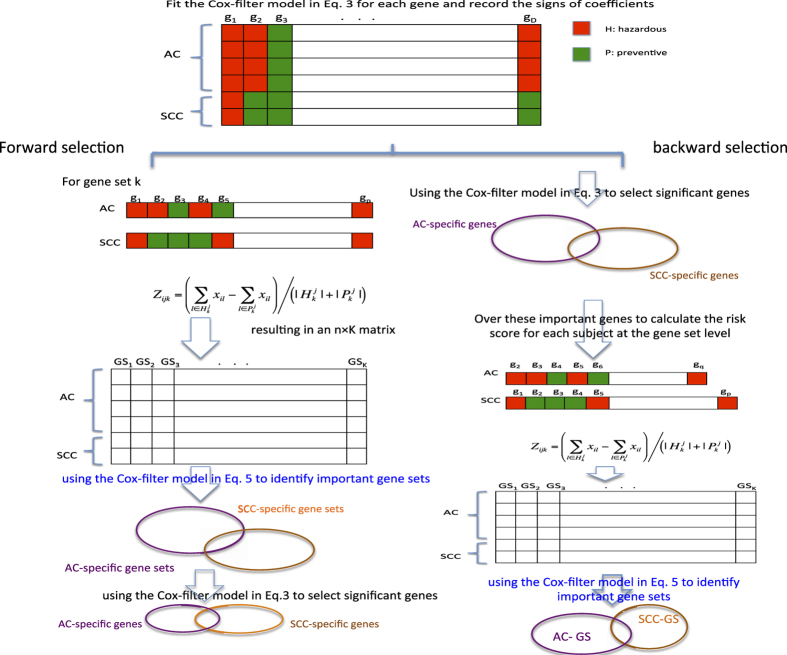
Graphic illustration of the proposed methods. (**A**) The forward Cox-filter method; (**B**) The backward Cox-filter method.

In both procedures, the adjusted p-values in Eq. 3 and Eq. 5 may be treated as tuning parameters. Over a grid of values, i.e., 0.01, 0.05, 0.1, 0.15 and 0.2, their optimal values are decided using 10-fold cross-validations. Figure 2 provides graphical elucidation of both the forward Cox-filter method and the backward Cox-filter method.

### Statistical Metrics

We use the censoring-adjusted C-statistic^24^ over the follow-up period (0,τ) to evaluate the performance of a resulting prognostic gene signature, where τ is a pre-specified time point. The C-statistic is defined as,


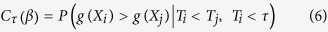


where g(X_i_) is the risk score for subject *i* with predictor vector X_i_ and T_i_ is the survival time of patient *i*.

The C-statistic can be interpreted as the probability of concordance between the predicted and observed survival times over all pairs of observations over the follow-up period (0,τ). Its asymptotic distribution is presented in the Appendix of the original paper^24^. Empirically, even though a value of 0.5 for the C-statistics corresponds to the random guess model, a prognostic signature with the C-statistic of relatively moderate values, i.e., between 0.6–0.7 is regarded to have satisfactory predictive performance^25^. The calculation of the C-statistic is implemented using the R survAUC package.

In addition, the Rand index is calculated to evaluate the stability or robustness of the resulting signatures. With k runs (e.g., the runs in an k-fold cross-validation or the applications of the method to k different data sets) of an algorithm, k lists of genes are obtained, i.e., gs_1_, gs_2_, … gs_k_. Then a Rand index is defined as


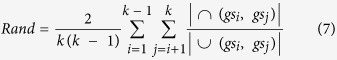


where ∩ represents the intersection between two gene lists, ∪ represents the union between the gene lists gs_i_ and gs_j_, and | | stands for the size of the corresponding set. The Rand index represents the agreement among the resulting signatures trained from different data sets.

With the gene lists being replaced by the pathway lists, the stability of resulting gene sets is evaluated using the Rand index.

### Statistical Language and Packages

All statistical analysis was carried out in the R language version 3.2 (www.r-project.org).

## Additional Information

**How to cite this article:** Tian, S. *et al*. Identification of prognostic genes and gene sets for early-stage non-small cell lung cancer using bi-level selection methods. *Sci. Rep.* **7**, 46164; doi: 10.1038/srep46164 (2017).

**Publisher's note:** Springer Nature remains neutral with regard to jurisdictional claims in published maps and institutional affiliations.

## Figures and Tables

**Table 1:** The results of simulation 1.

	Size	ARRB1(%)	ECE2(%)	COPA(%)	SMAD4(%)	C-Stat (SE)%	Rand (SE)%
Forward-AC	28.7	66	16	46	0	69.71(4.11)	34.68(5.56)
Forward-SCC	52.2	64	22	4	84	74.95(6.61)	28.02(2.26)
Backward-AC	49.5	0	16	40	0	66.99(3.10)	23.20(2.67)
Backward-SCC	60.4	0	44	76	54	62.65(5.55)	15.87(5.04)
Cox-filter: AC	59.2	100	4	0	0	54.09(7.09)	35.89(2.46)
Cox-filter: SCC	74.1	0	0	0	86	54.44(10.05)	26.47(2.80)
Cox-TGDR: AC	8.2	100	0	36	0	69.09(2.66)	32.13(3.33)
Cox-TGDR:SCC	3.8	100	0	4	0	53.34(7.79)	42.16(8.59)
LASSO: AC	37.8	100	94	0	2	76.00(2.20)	22.65(0.92)
LASSO: SCC	4.0	0	0	0	92	54.03(4.67)	34.05(7.75)

Note: Size: the average number of selected genes over 50 replicates. Under each gene symbol, its frequencies of being selected over 50 replicates by different algorithms are presented. Forward: forward Cox-filter selection; Backward: backward Cox-filter selection.

**Table 2:** The results of simulation 2.

	Size	ARRB1(%)	ECE2 (%)	COPA(%)	SMAD4(%)	C-Stat (SE) %	Rand (SE) %
Forward-AC	85.5	100	58	88	68	75.45(4.05)	44.44(2.76)
Forward-SCC	109.5	70	56	74	90	69.29(8.52)	38.58(2.41)
Backward-AC	110.4	64	76	92	66	72.39(4.04)	28.11(4.53)
Backward-SCC	142.3	54	56	84	86	70.62(7.28)	37.98(7.64)
Cox-filter: AC	78.9	100	20	0	74	72.31(2.38)	50.50(6.49)
Cox-filter: SCC	145.3	40	44	10	94	64.13(4.55)	21.32(8.11)
Cox-TGDR: AC	5.5	100	38	0	46	61.57(3.09)	35.03(5.43)
Cox-TGDR:SCC	8.7	98	64	22	90	54.16(4.61)	38.35(6.22)
LASSO: AC	28.7	98	72	48	98	81.27(2.10)	25.48(2.06)
LASSO: SCC	4.9	2	8	4	28	56.29(4.87)	21.35(1.27)

Note: Size: the average number of selected genes over 50 replicates. Under each gene symbol, its frequencies of being selected over 50 replicates by different algorithms are presented. Forward: forward Cox-filter selection; Backward: backward Cox-filter selection. C-Stat (SE): the mean of C-statistics over the replicates (its corresponding standard error).

**Table 3:** The frequencies for four causal genes under a random guess model.

	Size	ARRB1(%)	ECE2(%)	COPA(%)	SMAD4(%)
Forward-AC	2.46	0	0	0	0
Forward-SCC	8.64	0	0	4	0
Backward-AC	1.2	0	0	0	0
Backward-SCC	1.5	0	0	0	0

Note: Size: the average number of selected genes over 50 replicates. Under each gene symbol, its frequencies of being selected over 50 replicates by different algorithms are presented. Forward: forward Cox-filter selection; Backward: backward Cox-filter selection.

**Table 4:** Performance statistics for the NSCLC application.

Method: subtype	10-fold cross-validations		C Statistic
	Rand_gene (SE)	Rand_gs (SE)	Test set (SE)
A. Using the microarray data as the training set			
Forward: AC	38.33% (1.51%)	51.92% (4.17%)	66.82% (3.52%)
Forward: SCC	42.48% (1.13%)	69.89% (6.76%)	71.93% (4.38%)
Backward: AC	46.08% (0.66%)	43.45% (2.27%)	58.29% (3.42%)
Backward: SCC	46.04% (0.67%)	56.00% (2.99%)	52.80% (2.75%)
B. Using the RNA-Seq data as the training set			
Forward: AC	35.53% (0.67%)	57.38% (3.45%)	55.16% (5.98%)
Forward: SCC	36.15% (0.58%)	65.72% (1.91%)	66.25% (6.20%)
Backward: AC	40.89% (0.51%)	23.59% (5.21%)	57.28% (5.57%)
Backward: SCC	39.56% (0.55%)	27.03% (4.66%)	60.93% (7.07%)
C. Comparison with other relevant algorithms by training on the microarray data			
Cox-filter: AC	25.25% (3.68%)	—	60.34% (2.85%)
Cox-filter: SCC	24.75% (3.65%)	—	59.94% (2.55%)
Cox-TGDR: AC	17.07% (3.31%)	—	52.32% (5.49%)
Cox-TGDR: SCC	18.65% (5.33%)	—	48.83% (4.34%)
Lasso: AC^s^	23.97% (2.06%)	—	55.35% (6.78%)
Lasso: SCC^s^	23.77% (3.47%)	—	50.00% (7.88%)

Note: Rand_gene: the rand index which evaluates the stability at the gene level; Rand_gs: the rand index which evaluates the stability at the gene set level; Forward: forward Cox-filter selection; Backward: backward Cox-filter selection; –: not available as the method only can carry out gene set-level selection. ^S^separately on each subtype because the method itself does not account for subtype information; SE: the standard errors obtained using the bootstrapped samples. In last column, the C-statistics and their standard errors for different methods on the test set are listed.

**Table 5:** Adjusted prognostic values of the resulting signatures in present of other clinical factors.

	Forward: AC	Forward: SCC	Backward: AC	Backward: SCC
	β (p-value)	β (p-value)	β (p-value)	β (p-value)
Signature (risk score)	1.6(9.2×10^−7^)*	1.85(0.02)*	3.01(3.4×10^−6^)*	4.24(0.02)*
Female versus male	−0.27(0.39)	−0.21(0.99)	−0.23(0.46)	−0.21(0.99)
Age	0.03(0.08)	0.04(0.37)	0.03(0.11)	0.02(0.69)
Smoking vs non-smoking	0.24(0.47)	−2.18(0.05)*	0.26(0.43)	−1.91(0.08)

Note: β: the estimated coefficient values in the multivariate Cox regression model using the prognostic signature, age, sex, and smoking status as covariates, representing the log hazard ratio. *p-value < 0.05, which is regarded as to be statistical significance.
